# Role for a Filamentous Nuclear Assembly of IFI16, DNA, and Host Factors in Restriction of Herpesviral Infection

**DOI:** 10.1128/mBio.02621-18

**Published:** 2019-01-22

**Authors:** Philipp E. Merkl, David M. Knipe

**Affiliations:** a Department of Microbiology, Harvard Medical School, Boston, Massachusetts, USA; Virginia Polytechnic Institute and State University; University of North Carolina-Chapel Hill; Wistar Institute

**Keywords:** DNA virus, chromatin, epigenetics, signaling, supramolecular organizing center

## Abstract

Several host cell nuclear factors are known to restrict herpes simplex virus 1 (HSV-1) replication, but their mechanisms of action remain to be defined. Interferon-inducible protein 16 (IFI16) and the nuclear domain 10-associated proteins, such as promyelocytic leukemia (PML) protein, localize to input viral genomes, but they are also capable of restricting progeny viral transcription. In this study, we used structured illumination microscopy to show that after HSV DNA replication, IFI16 forms nuclear filamentous structures on DNA within a subset of nuclear replication compartments in HSV-1 ICP0-null mutant virus-infected human cells. The ability to form filaments in different cell types correlates with the efficiency of restriction, and the kinetics of filament formation and epigenetic changes are similar. Thus, both are consistent with the filamentous structures being involved in epigenetic silencing of viral progeny DNA. IFI16 filaments recruit other restriction factors, including PML, Sp100, and ATRX, to aid in the restriction. Although the filaments are only in a subset of the replication compartments, IFI16 reduces the levels of elongation-competent RNA polymerase II (Pol II) in all replication compartments. Therefore, we propose that IFI16 filaments with associated restriction factors that form in replication compartments constitute a “restrictosome” structure that signals in *cis* and *trans* to silence the progeny viral DNA throughout the infected cell nucleus. The IFI16 filamentous structure may constitute the first known nuclear supramolecular organizing center for signaling in the cell nucleus.

## Abstract (precis)

Mammalian cells exhibit numerous strategies to recognize and contain viral infections. The best-characterized antiviral responses are those that are induced within the cytosol by receptors that activate interferon responses or shut down translation. Antiviral responses also occur in the nucleus, yet these intranuclear innate immune responses are poorly defined at the receptor-proximal level. In this study, we explored the ability of cells to restrict infection by assembling viral DNA into transcriptionally silent heterochromatin within the nucleus. We found that the IFI16 restriction factor forms filaments on DNA within infected cells. These filaments recruit antiviral restriction factors to prevent viral replication in various cell types. Mechanistically, IFI16 filaments inhibit the recruitment of RNA polymerase II to viral genes. We propose that IFI16 filaments with associated restriction factors constitute a “restrictosome” structure that can signal to other parts of the nucleus where foreign DNA is located that it should be silenced.

## INTRODUCTION

Central to the antiviral defense networks of human cells are receptors that detect viral nucleic acids. These receptors are numerous and can display specificity for DNA or RNA in a sequence-dependent or -independent manner. Studies over the last decade have demonstrated that the cytosol is a common subcellular location of antiviral receptors, as the proteins cGAS, RIG-I, and MDA5 survey this compartment for viral nucleic acids. Upon binding to viral DNA or RNA, all of these receptors induce the transcription of genes encoding cytokines and interferons (IFNs), the actions of which constitute a potent antiviral response. However, the cytosol is not the only site of DNA or RNA detection—cells also survey the intralumenal space of endosomes and the intranuclear space for viral nucleic acids. Within the nucleus, viral DNA is sensed and loaded with heterochromatin to silence its expression and restrict viral replication (1, 2). Several restriction factors, including PML, ATRX, DAXX, and IFI16, limit herpesviral gene expression and replication (3–5). These cellular factors are thought to bind to incoming viral DNA and recruit cellular epigenetic silencing factors that assemble heterochromatin on the viral DNA (5, 6) or to assemble a structure around the DNA that might block access of transcription factors and RNA polymerase II (Pol II) to prevent its expression (7). Of these, IFI16 was first reported as a sensor of transfected and viral DNA involved in innate signaling and epigenetic silencing of viral DNA or plasmid DNA not delivered in nucleosomes (5, 6, 8–11). IFI16 binds cooperatively to naked single- and double-stranded DNA in a length-dependent manner (12) mediated by its HIN domains via the sugar-phosphate backbone in a sequence-unspecific fashion with a footprint of about 8 bases (13, 14). IFI16-DNA complex formation *in vitro* involves initial binding of IFI16, followed by one-dimensional diffusion along the DNA substrate (15). This diffusion leads to IFI16-IFI16 encounters and results in cluster formation. Four IFI16 copies are required to initiate immobile cluster assembly, with an optimally stable cluster consisting of 10 IFI16 protomers (15). The presence of nucleosomes on the DNA prevented IFI16 diffusion and multimerization (15), providing a basis for IFI16 discrimination between foreign, unchromatinized DNA and cellular chromatin.

Further evidence of the importance of IFI16 and the PML nuclear body proteins in limiting herpes simplex viral replication is that HSV has evolved the ICP0 protein to promote the degradation of the PML, IFI16, ATRX, and Sp100 proteins and prevent their restriction activities (4, 8, 16, 17). Therefore, ICP0-null mutant viruses are used to detect the full restrictive capacity of these host proteins. Depletion of IFI16 by knockdown or knockout leads to increased replication of ICP0-deficient viruses (5, 6) due to increased viral protein expression and decreased viral heterochromatin.

Our recent study demonstrated that IFI16 acts on both parental and progeny viral DNA of ICP0-null viruses to reduce immediate early (IE) gene expression (18). IFI16 localizes to parental viral genome complexes in the infected cell nucleus at very early times after infection (8, 11, 19–21), and we have hypothesized that IFI16 binds to the input parental DNA and recruits epigenetic silencing factors to the viral genomes (1, 2). However, it remains unclear how IFI16 functions to restrict transcription from progeny viral genomes. HSV DNA replication occurs throughout globular replication compartments (RCs) within the nucleus of infected cells (22–24), and individual RCs originate from amplification of one input viral genome (25), which then fuse (26, 27). In ICP0^−^ virus-infected cells, we found that cells with larger RCs showed accumulation of IFI16 within those compartments (5), and others found IFI16 in thread-like structures (19). Thus, IFI16 appeared to not colocalize with all of the progeny viral DNA in RCs.

IFI16 has been shown to form filaments on DNA *in vitro*, and electron microscopic analysis of these filaments defined a width for them of 20 to 25 nm (15). We hypothesized that the infected cell IFI16 filaments could be assembled on viral progeny DNA *in vivo*. Superresolution microscopy showed that the IFI16 filaments are common in at least one replication compartment per cell and that PML, Sp100, and to a lesser extent ATRX assemble on the filaments. Not all replication compartments contain these filaments, but Pol II recruitment is reduced in all compartments equally, whether they have IFI16 or not. Based on these and other results, we hypothesize that these filaments are “restrictosomes” that signal in *cis* and in *trans* to other parts of the infected cell nucleus to restrict transcription from other viral genomes.

## RESULTS

### IFI16 forms filaments in a subset of RCs.

**FIG 1:**
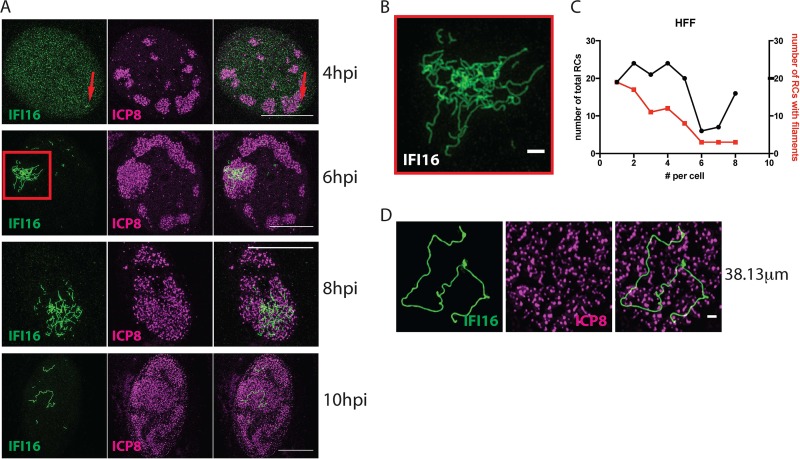
IFI16 forms filamentous structures in replication compartments in cells infected with an HSV-1 ICP0-null virus. HFF cells were infected with 7134 virus at an MOI of 5. (A) Cells were fixed at 4, 6, 8, and 10 hpi and immunostained for IFI16 (green) and ICP8 (magenta). Images show nuclei of respective cells at indicated times postinfection. The scale bar represents 10 µm. *n* ≥ 3 experiments. (B) Enlarged section of the 6-hpi time point, indicated by the red quadrangle. (C) Frequency distributions of total RCs and RCs with filaments in a population of cells (≥50 cells per sample). (D) Single IFI16 filament (green) in an RC in a nucleus of a cell at the edge of a plaque. The scale bar length is 1 µm.

IFI16 restricts expression of HSV-1 gene expression from both input and progeny genomes (18), but it was unclear how IFI16 could restrict expression from viral progeny DNA genomes. To further define the localization of IFI16 at times when it is restricting viral gene expression from progeny DNA, we infected human foreskin fibroblasts (HFFs) with an ICP0-deficient recombinant strain, HSV-1 7134. At various times after infection, we performed structured illumination microscopy (SIM) to detect endogenous IFI16. We observed that small filamentous IFI16 structures appeared in replication compartments (RCs) as early as 4 h postinfection (hpi) (Fig. 1A, red arrows). By 6 hpi, large dense filamentous networks of IFI16 were observed in a subset of replication compartments with increasing RC size (Fig. 1A and B), and the IFI16 structures became less compact by 8 hpi (Fig. 1A). By 10 hpi, the large filament networks were diminished, consistent with the short half-life of IFI16 and decreasing levels of IFI16 observed over time in 7134 virus-infected cells using immunoblotting (28).

To determine the frequency of the IFI16 filaments, we measured IFI16 filament formation under a variety of experimental conditions, including cell density, stage of infection, and multiplicity of infection (MOI). Under all conditions examined, we observed a correlation between the frequency of RCs, viral ICP8 expression, and IFI16 filament formation (see Fig. S1A to C in the supplemental material). The percentage of cells containing IFI16 filaments reached almost 100% under certain conditions, such as an MOI of 100 or high cell density. The IFI16 filaments were observed in only a subset of the replication compartments within any given cell as the numbers of compartments with filaments per cell were significantly lower than the numbers of replication compartments per cell (Fig. 1C; *P* = 0.04504, Mann-Whitney-Wilcoxon test).

10.1128/mBio.02621-18.1 FIG S1 Formation of IFI16 filaments under various conditions. Shown is the immunofluorescence time course in 7134-infected HFF cells’ PML (green) and ICP8 (magenta) 4 to 10 hpi. (A to C) HFF cells were seeded at 0.25 × 10^5^ or 1 × 10^5^ cells per well in a 24-well plate. Infection with 7134 virus was done at MOI of 1, 10, or 100. Samples were fixed at 6, 8, or 10 hpi. (A) ICP8^+^ cells were counted. (B) Among the ICP8^+^ cells, RC^+^ cells were counted. (C) Among the RC^+^ cells, filament-positive cells were counted. (D) Confluent HFF cells were infected with 7134 virus at 0.5 PFU/cell. Cells were fixed after plaques formed and then were immunostained for IFI16 (green) and ICP8 (magenta). Two white lines denote the approximate border between the plaque and surrounding infected cells as well as the border between the infected cells and uninfected cells. Red arrows indicate the spread of infection from the plaque. The scale bar length is 100 µm. (E) Infected cells at the edge of the plaque. The scale bar length is 100 µm. (F) Single infected cell with large network of IFI16 filaments. The scale bar length is 10 µm. *n* = 2 experiments. Download FIG S1, PDF file, 2.1 MB. Copyright © 2019 Merkl et al. 2019 Merkl et al. This content is distributed under the terms of the Creative Commons Attribution 4.0 International license.

To determine if the time-dependent changes in IFI16 filament formation reflect events that occur during a natural multicell infectious process, we studied IFI16 filament formation in cells surrounding a plaque after infection with a low MOI. This procedure models cell-to-cell spreading of HSV-1 in human tissue (Fig. S1D to F). The distance from the center of the plaque should correlate with the time of infection, with cells closest to the plaque being infected for longer times than cells furthest from the center. We found that infected (ICP8^+^) cells furthest from the plaque displayed the most intense staining of IFI16 filaments, whereas cells closest to the center contained minimal or no filaments (Fig. S1D and E). Thus, in a cell culture model of viral spread, IFI16 filament formation occurs in a dynamic and time-dependent fashion.

Examination of individual infected cells revealed that IFI16 filaments demonstrated length heterogeneity. Occasionally we observed one long filament in the nucleus of the cell (Fig. 1D), with maximum observed lengths of around 38 µm (Fig. S1F). Given that the HSV-1 genome has a length of approximately 152 kbp and based on the fact that 1 bp of B-DNA would be approximately 340 pm, the theoretical length of the HSV-1 genome as B-DNA would be 52 µm. Considering the possible compaction of the DNA and the fact that we measured only the length of a 2-dimensional projection instead of the actual 3-dimensional object, the length of some single filaments approximated that of a viral genome. Considering that one IFI16 protein occupies 15 bp (12), such a filament would harbor roughly 10,000 IFI16 molecules, if uniformly coated.

### Viral DNA synthesis is required for IFI16 filament formation *in vivo*.

**FIG 2:**
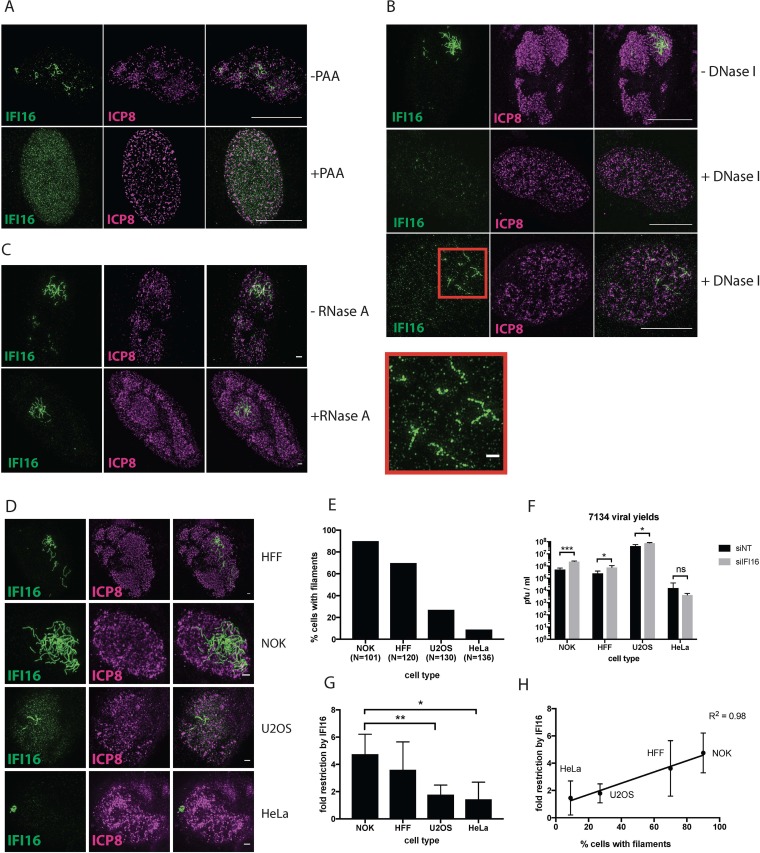
Role of viral DNA synthesis and human cell type in IFI16 filament formation. (A) HFF cells were infected with 7134 virus at an MOI of 5 in the presence or absence of PAA. Cells were fixed at 6 hpi, and immunostaining for IFI16 and ICP8 was performed. The scale bar length is 10 µm. (B) HFF cells were infected with 7134 virus at an MOI of 5. Mild fixation and DNase I treatment were performed at 6 hpi as outlined in Materials and Methods. Cells were then fixed and immunostained for IFI16 (green) and ICP8 (magenta). The enlarged section is denoted by a red quadrangle. Scale bar lengths in montages are 10 µm and 1 µm in the enlarged section. (C) As in panel B, but with RNase A treatment. (D) HFF, NOK, U2OS, and HeLa cells were infected with 7134 virus at an MOI of 5. Cells were fixed 6 hpi and immunostained for IFI16/ICP8. Panels A to D represent results from 2 experiments. (E) Cells were counted after immunostaining, and the percentages of cells with IFI16 filaments are shown for each cell type. (F) HFF, NOK, U2OS, and HeLa cells were treated with nontargeting or IFI16-specific siRNAs and infected with 7134 virus at an MOI of 0.1. Progeny viruses were harvested at 48 hpi, titrated on U2OS cells, and plotted as PFU/ml. Statistical analysis between columns was done via *t* test. (G) Fold restriction by IFI16 was calculated by dividing viral yields in the absence of IFI16 by viral yields in the presence of IFI16 and plotted for each cell type. Statistical analysis was done by *t* test. (H) Values obtained in panels F and G were plotted against each other. Linear regression analysis was performed with Prism.

Based on our initial observation that IFI16 filaments emerge at similar times as replication compartments, we hypothesized that filament formation might be linked to viral DNA (vDNA) replication. To address the role of input and progeny vDNA in formation of IFI16 filaments, we infected HFFs in the presence or absence of phosphonoacetic acid (PAA), an inhibitor of the HSV DNA polymerase. ICP8 formed prereplicative sites in the presence of PAA, confirming that PAA had inhibited viral DNA replication (22) (Fig. 2A). While IFI16 filaments formed in infected cells in the absence of PAA (Fig. 2A), PAA treatment prevented IFI16 filament formation. Indeed, in infected cells, PAA treatment rendered IFI16 localization patterns similar to those of uninfected cells (Fig. 2A). To determine if the presence of nucleic acids in the nucleus influenced the stability of IFI16 filament, we briefly fixed infected cells and treated them with DNase I or RNase A. Upon DNase treatment, IFI16 filaments were either completely degraded, resulting in diffuse IFI16 staining, or were fragmented (Fig. 2B). In contrast, RNase A digestion did not alter the abundance, shape, or morphology of IFI16 filaments (Fig. 2C). These results showed that the IFI16 filaments form on DNA, likely viral progeny DNA, because viral DNA synthesis is required for IFI16 filament formation.

### Levels of viral restriction correlated with frequency of filament formation in different human cell types.

To determine if there was a relationship between the IFI16 filaments and the restrictive effect exerted by IFI16, we measured IFI16 filament formation and IFI16-mediated restriction of ICP0^−^ virus replication in different cell types. Thus, we infected normal oral keratinocyte (NOK), HFF, U2OS and HeLa cells with 7134 virus, fixed the cells at 6 hpi, and immunostained for IFI16 and ICP8. Quantitation of cells bearing IFI16 filaments showed that NOK cells had the highest percentage of nuclei with filament-containing RCs (90%), followed by HFF (70%), U2OS (27%) and HeLa (9%) cells (Fig. 2D and E). To elucidate the restrictive effect of IFI16 in these cell types, we treated them with either nontargeting or IFI16-specific small interfering RNAs (siRNAs) and infected them with 7134 virus at an MOI of 0.1. Viral yields increased significantly due to IFI16 depletion in NOK, HFF, and U2OS cells, confirming previous results, but were largely unaffected in HeLa cells (Fig. 2F). Restriction was highest in NOK cells (4.7-fold), followed by HFF (3.6-fold), U2OS (1.8-fold), and HeLa (1.4-fold) cells (Fig. 2G). Linear regression analysis of the percentage of nuclei with filaments versus fold restriction showed a clear correlation (Fig. 2H; *R*^2^ = 0.98). We therefore concluded that formation of IFI16 filaments correlated with and was related to IFI16 restriction of replication of HSV-1 ICP0^−^ viruses.

### Kinetics of the IFI16 effects on viral chromatin.

**FIG 3:**
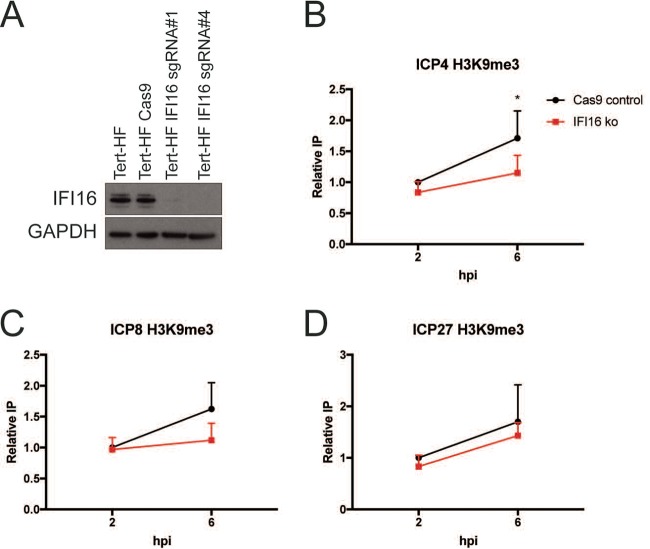
Formation of IFI16 filaments is accompanied by epigenetic silencing of progeny vDNA. (A) Western blot confirmation of lack of expression of IFI16 in Cas9 control or IFI16ko Tert-HF cells. (B to D) Chromatin immunoprecipitation after 7134 virus infection of Cas9 or IFI16ko cells was performed with antibodies specific for the H3K9me3. Results were plotted as percentage of immunoprecipitated protein normalized to the 2-h Cas9 control value for each experiment (*n* = 3). (B) *ICP4* gene. (C) *ICP8* gene. (D) *ICP27* gene. *, *P* < 0.5, by unpaired two-tailed *t* test. Error bars represent standard deviation (SD).

To investigate the kinetics of IFI16 effects on chromatin relative to filament formation, we examined chromatin composition on selected viral genes in the presence or absence of IFI16. For these studies, we generated IFI16 knockout and control Cas9 cells in telomerase reverse transcriptase (TERT)-immortalized human fibroblasts (Tert-HF cells) as described in Materials and Methods. The guide RNA (gRNA) 4 IFI16 knockout (IFI16ko) cells showed a complete absence of IFI16 (Fig. 3A), so they were used in these studies. When chromatin immunoprecipitations were performed, we observed that depletion of IFI16 led to a statistically significant decrease in the H3K9me3 heterochromatin mark on the *ICP4* gene at 6 hpi (*P* = 0.4) (Fig. 3B) and consistent with previous results (5) led to an H3K9me3 decrease that approached statistical significance on the *ICP8* gene at 6 hpi (*P* = 0.5) (Fig. 3C), but did not significantly change H3K9me3 on the *ICP27* gene (*P* = 0.43) (Fig. 3D). Because there was no effect on H3K9me3 levels at 2 hpi (Fig. 3B to D), the effect of IFI16 on heterochromatin appeared at approximately the same time as IFI16 filaments were formed, providing further evidence that the IFI16 filaments were related to restriction of HSV-1.

### Localization of RNA Pol II to RCs with or without IFI16.

**FIG 4:**
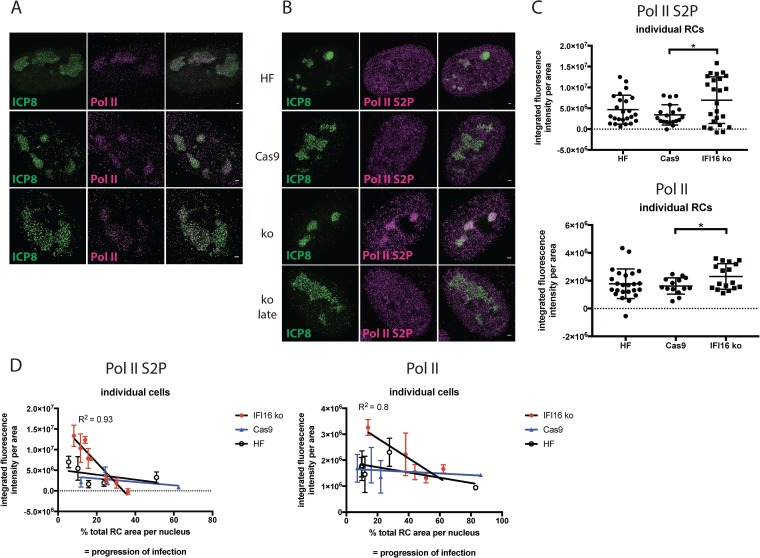
Levels of elongation-competent and initiation-competent RNA polymerase II are enhanced in replication compartments in the absence of IFI16. (A and B) Tert-HF, Cas9, and IFI16ko cells were infected with 7134 virus and fixed at 6 hpi. Immunostaining was done for ICP8/Pol II or ICP8/Pol II S2P. *n* = 3 experiments. (C) Pol II S2P or Pol II signal intensities per area in individual replication compartments were determined and compared for different cell lines. Statistical analysis was done with a *t* test. (D) Pol II S2P signal intensities per area in individual replication compartments of individual cells were determined, averaged, and plotted against the total relative replication compartment area of the respective cell. Statistical analysis was done with a *t* test.

Viral genes are transcribed by cellular Pol II in RCs after viral DNA synthesis (29–33); thus, the recruitment of Pol II may be affected by IFI16-mediated silencing. Initiation-competent Pol II is associated with an unphosphorylated C-terminal domain (CTD), whereas phosphorylation at serine 2 (S2P) is a hallmark of elongation-competent Pol II. Unphosphorylated Pol II and Pol II S2P localize in HSV replication compartments (30). To determine whether IFI16 influenced RNA Pol II recruitment, we infected IFI16 knockout (IFI16ko) and Cas9 control cells and immunostained for combinations of ICP8/Pol II or ICP8/Pol II S2P. We confirmed previous observations (30, 34) that RNA Pol II is concentrated in RCs of infected cells (Fig. 4A). Enhanced resolution with SIM showed that ICP8 and Pol II foci did not colocalize (absence of white color in merge) but occupied different spaces within RCs (Fig. 4A), implying spatial separation of vDNA replication and transcription, as we reported previously for the HSV ICP4 transcriptional activator and ICP8 DNA replication protein (24). No obvious differences in Pol II or Pol II S2P staining levels were observed in the proximity of IFI16 filaments (see Fig. S2 in the supplemental material). However, Pol II S2P signal was visibly enhanced in early or smaller RCs in the absence of IFI16 (Fig. 4D). At later stages of infection, this enhancement seemed to diminish visually (Fig. 4B), consistent with loss of Pol II at later times of infection (35). To assess these findings quantitatively, we measured Pol II or Pol II S2P fluorescence signal intensities per area in individual RCs of multiple cells. We observed that total Pol II and Pol II S2P accumulation in RCs was significantly enhanced by 2- and 1.5-fold in IFI16ko cells compared to that in Cas9 or HF cells, respectively (Fig. 4C). To measure Pol II signal intensity as a function of the stage of infection, we measured Pol II or Pol II S2P signal intensities per area of all RCs within single nuclei versus the combined RC area expressed as percentage of the area of the respective nucleus to serve as a measure of progression of infection (Fig. 4D). Pol II and Pol II S2P signal intensities in different RCs within a nucleus were comparable, as shown visibly in the images in Fig. S2 and as documented by the narrow range of error bars, independent of whether filaments were present in the respective RCs or not (Fig. 4D). For both Pol II and Pol II S2P, we observed increased average signal intensity in the absence of IFI16 in nuclei with low relative RC coverage (Fig. 4D). At later stages during infection (i.e., between 30% [Pol II S2P] or 50% [Pol II] coverage), signal intensities became lower and comparable with or without IFI16. Regression analysis showed that the slopes of the curves were significantly different in the IFI16ko cells compared to the Cas9 control cells or HF control cells. We concluded that the absence of IFI16 leads to enhanced recruitment of both total and S2P RNA Pol II. These findings are most notable when considering our observation that IFI16 filaments were not associated with every RC present within an infected cell nucleus, as described above. It is therefore possible that IFI16 filaments operate as a signal transduction center to inhibit Pol II accumulation in or recruitment to RCs throughout the infected cell nucleus.

10.1128/mBio.02621-18.2 FIG S2 IFI16 and Pol II/Pol II S2P immunofluorescence. HFF cells were infected with 7134 virus, fixed at 6 hpi, and immunostained with antibodies specific for IFI16 (green) and Pol II (magenta) or IFI16 (green) and Pol II S2P (magenta). *n* = 3 experiments. Download FIG S2, PDF file, 0.6 MB. Copyright © 2019 Merkl et al. 2019 Merkl et al. This content is distributed under the terms of the Creative Commons Attribution 4.0 International license.

### PML, Sp100, and ATRX colocalize with IFI16 filaments.

Based on the above indication that IFI16 filaments may operate as a signaling hub to interfere with Pol II activity on viral genes throughout the nucleus, we reasoned that these filaments may recruit other proteins involved in viral restriction. This idea is consistent with the emerging recognition that innate immune signaling pathways are activated by filament-based supramolecular organizing centers (SMOCs) (36). These SMOCs contain several proteins that act together to drive inflammation and host defense. However, all of our knowledge of SMOC biology relates to protein complexes in the cytosol. We therefore considered whether IFI16 filaments might represent an example of a filament-based SMOC that operates in the nucleus.

**FIG 5:**
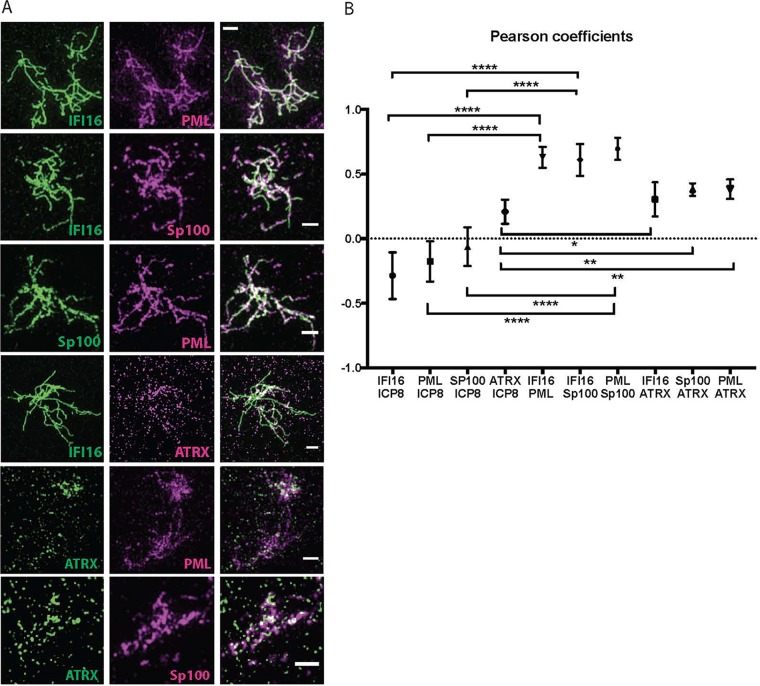
Other host restriction factors colocalize with IFI16 filaments. (A) HFF cells were infected with 7134 virus at an MOI of 5 and fixed at 6 hpi. Immunostaining was performed for all dual combinations of IFI16, PML, Sp100, and ATRX antibodies. The scale bar length is 1 µm. *n* = 3 experiments. (B) Colocalization analysis for all double combinations of antibodies specific for IFI16, PML, Sp100, ATRX, and ICP8 was performed with Fiji as described in Materials and Methods.

The cellular proteins PML, Sp100, and ATRX, like IFI16, are involved in viral DNA silencing, and their degradation is promoted by the HSV ICP0 protein. While the genes encoding these factors are necessary for the same antiviral process, the relationship between the proteins within the nucleus is unclear. To determine whether other proteins were associated with the IFI16 filaments, we performed costaining with antibodies specific for IFI16, HSV ICP8, or the cellular proteins PML, SP100, and ATRX. Dual-staining experiments with antibodies specific for IFI16/PML, IFI16/Sp100, or PML/Sp100 showed strong visual colocalization between the filaments formed by the three proteins (Fig. 5A). This finding was confirmed quantitatively by determination of Pearson coefficients, demonstrating strong colocalization between IFI16, Sp100, and PML (Fig. 5B). No colocalization but perhaps weak anticolocalization between these three proteins and ICP8 was observed (Fig. 5B; see Fig. S3A and B in the supplemental material). ATRX staining was found to partially colocalize with the IFI16 filaments in that ATRX punctate colocalized with the filaments (Fig. 5A), resulting in an intermediate Pearson coefficient (Fig. 5B).

10.1128/mBio.02621-18.3 FIG S3 PML and Sp100 filaments in HFF cells and 3D iso-surface rendering of IFI16 filaments. (A and B) Dual immunostaining with antibodies specific for PML/ICP8 or Sp100/ICP8 in HFF cells after infection with 7134 virus (MOI of 5, 6 hpi). *n* = 3 experiments. (C) Imaris iso-surface rendering of the original image displayed in Fig. 1A from different angles. Download FIG S3, PDF file, 2.1 MB. Copyright © 2019 Merkl et al. 2019 Merkl et al. This content is distributed under the terms of the Creative Commons Attribution 4.0 International license.

Three-dimensional iso-surface renderings of IFI16 filaments and ICP8 also supported the hypothesis that the filaments were not generally colocalizing with ICP8 (see Fig. S3C and Movie S1 in the supplemental material). Thus, four antiviral proteins, IFI16, PML, Sp100, and ATRX, colocalize within filaments in replication compartments.

10.1128/mBio.02621-18.5 MOVIE S1 Intranuclear structures of IFI16 (green) and ICP8 (magenta) are shown. The movie represents a stack of 21 images with a total thickness of 2.5 μm in the z dimension. Actual fluorescence data are shown in the beginning, followed by a three-dimensional iso-surface rendering. The rendering and movie were generated with Imaris software. Download Movie S1, MP4 file, 3.4 MB. Copyright © 2019 Merkl et al. 2019 Merkl et al. This content is distributed under the terms of the Creative Commons Attribution 4.0 International license.

### IFI16 is necessary for the filamentous association of PML and Sp100.

**FIG 6:**
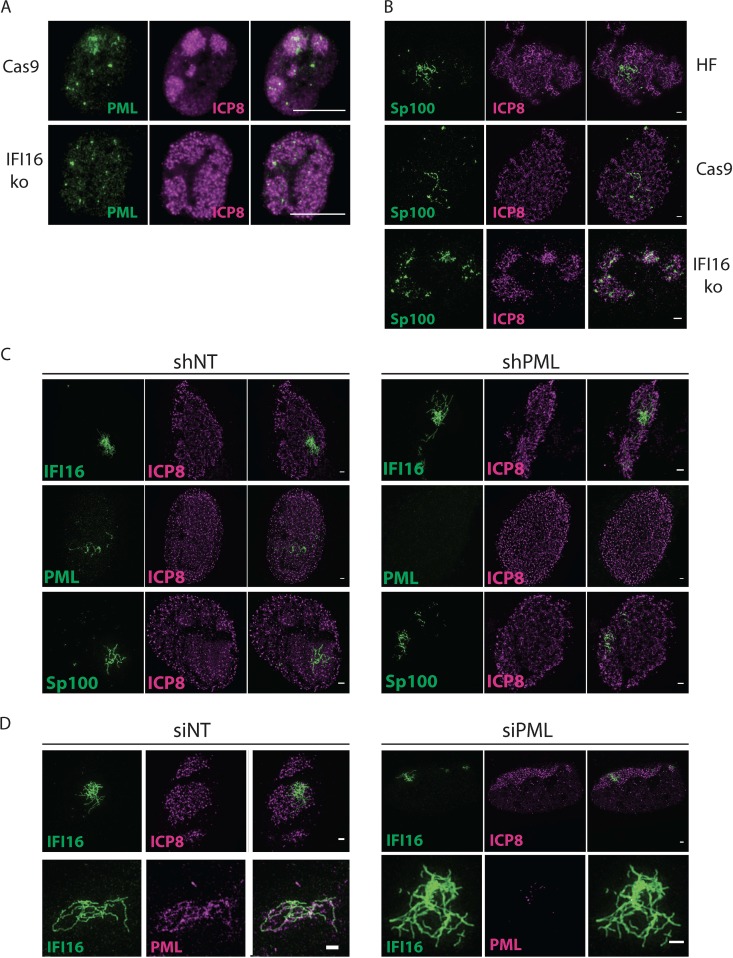
IFI16 organizes filamentous aggregation of ND-10 proteins. (A) Cas9 control and IFI16ko cells were infected with 7134 virus at an MOI of 5 and fixed at 6 hpi. Immunostaining was performed for PML (green) and ICP8 (magenta). The scale bar length is 10 µm. Images were taken with a Zeiss Axioplan microscope. (B) Tert-HF and Tert-HF-derived Cas9 and IFI16ko cells were infected with 7134 virus and processed as in panel A. Immunostaining was done for Sp100 (green) and ICP8 (magenta). (C) HFF cells stably expressing nontargeting or PML-specific shRNAs were infected with 7134 virus and processed as in panel A. Immunostaining was performed for IFI16/ICP8, PML/ICP8, or Sp100/ICP8. Scale bars represent 1 µm. (D) HFF cells were treated with nontargeting or PML-specific siRNAs, infected with 7134 virus, and processed as in panel A. Immunostaining was performed for IFI16/ICP8 or IFI16/PML. Scale bars represent 1 µm. *n* = 2 experiments.

To determine if IFI16 was required for the filamentous localization patterns of the PML and Sp100 proteins, we infected cells in which the *IFI16* gene had been knocked out via CRISPR/Cas9 (18) or control HFF cells that only expressed Cas9 with 7134 virus and immunostained for ICP8 and PML. In IFI16ko cells, we did not observe any PML filaments or structures beyond the puncta already present in mock-infected cells (Fig. 6A; compare Fig. S4A and S4B in the supplemental material). Experiments using siRNA-mediated knockdown of IFI16 showed the same result (not shown). Using the IFI16ko Tert-HF cells described above, we did not detect Sp100 filaments or oligomerization besides the ND10 punctate staining (Fig. 6B; Fig. S4C). However, there seemed to be recruitment of Sp100 to replication compartments in the absence of IFI16.

10.1128/mBio.02621-18.4 FIG S4 PML and Sp100 localization in IFI16ko cells. **(**A and B) Time course of PML (green) and ICP8 (magenta) localization in Cas9 and IFI16ko cells after infection with 7134 virus (MOI of 5). (C) Immunofluorescence images showing IFI16 (green) and Sp100 (magenta) in HF, Cas9, and IFI16ko cells after infection with 7134 virus (MOI of 5). *n* = 3 experiments. Download FIG S4, PDF file, 0.8 MB. Copyright © 2019 Merkl et al. 2019 Merkl et al. This content is distributed under the terms of the Creative Commons Attribution 4.0 International license.

In addition, we determined if IFI16 could form filaments in the absence of PML. We employed two strategies as described previously (18), in which PML was depleted by siRNA or short hairpin RNA (shRNA). Neither shRNA nor siRNA depletion of PML affected IFI16 filament formation (Fig. 6C and D). Sp100 showed filamentous aggregation in the shPML- and siPML-depleted cells. Based on these findings, we propose that IFI16 binding to viral DNA induces the assembly of an IFI16-dependent SMOC consisting of PML and Sp100, which collectively operate to silence HSV DNA.

## DISCUSSION

Filament-based supramolecular organizing centers (SMOCs) are known to form in the cytoplasm as a consequence of recognition of viral RNA and to initiate innate signaling pathways. However, little is known about the organization of innate immune and intrinsic resistance pathways in the infected cell nucleus. In this study, we have found that IFI16, DNA, and other host restriction factors form nuclear filamentous structures that appear to function to signal to progeny viral genomes throughout the infected cell nucleus to effect their epigenetic silencing and reduce RNA Pol II recruitment to the viral genes. Initiation of the filaments involves IFI16 binding to DNA, likely progeny viral DNA, to nucleate formation of filaments as a result of the highly cooperative nature of IFI16 binding to DNA. IFI16 then recruits in effector molecules, including the host restriction factors PML, Sp100, and ATRX. Formation of the filaments shows a strong correlation with restriction of replication of an HSV-1 mutant viral strain. Therefore, the IFI16 filamentous structures may provide the first example of an SMOC that is assembled and functions in the cell nucleus.

Cellular factors that restrict DNA virus gene expression in the nuclei of infected cells are thought to localize to input viral DNA genomes and reduce initial viral transcription, but how these factors can reduce viral gene expression from progeny viral genomes that are amplified by 1,000-fold or more is not known. In this study, we found that the IFI16 restriction factor forms filamentous structures in a subset of the HSV nuclear replication compartments where viral DNA replication, late gene transcription, and DNA encapsidation take place (37). Other cellular restriction factors, including PML, Sp100, and ATRX, are recruited to the filamentous structures, and lamin B may serve to anchor the filaments in the nucleus (results not shown). These filamentous structures are notable in not colocalizing with the bulk of the progeny viral DNA molecules. IFI16 reduces the expression of viral genes from both input and progeny viral genomes (18), and evidence presented here shows a correlation between the ability of different human cell types to form IFI16 filaments and the ability of IFI16 in that cell type to restrict HSV-1 ICP0^−^ mutant virus gene expression and replication. Because the majority of IFI16 in these cells at later times is in the filaments but RNA Pol II is recruited uniformly throughout the replication compartment and even in compartments without IFI16 filaments, we hypothesize that the IFI16 filaments with associated host restriction factors can send signals to viral progeny DNA molecules throughout the nucleus that reduce transcription of the viral genomes. Supramolecular assemblies are believed to drive the signaling in inflammasomes, death-induced signaling complexes, MAVS-RIG-I signaling assemblies, and Myddosome activation of the Toll-like receptor (TLR) pathway (36). We speculate that the IFI16 filamentous assembly is a similar signaling platform that recognizes individual progeny DNA molecule and signals in *trans* to all of the viral DNA molecules in the infected cell nucleus. We speculate below on possible mechanisms for this mechanism.

Previous studies had found that IFI16 forms aggregate or fibrous structures in the nuclei of cells infected with an ICP0-null virus (5, 19, 20). These results were supported by *in vitro* studies (12) showing that IFI16 could form filaments on DNA. Our results demonstrate that IFI16, PML, and Sp100 form colocalizing filamentous structures in RCs in nuclei of infected cells. Diner et al. (11) had previously observed interactions between IFI16 and ND10 proteins in infected cells, but it is unclear if the filaments would have been solubilized under their conditions. The filaments emerge at the time of onset of vDNA replication by the 7134 ICP0^−^ virus (18) and are putatively formed on newly synthesized vDNA. Hereby, IFI16 is required for recruitment of the other factors. IFI16/PML/Sp100 filaments appear to be tethered to the nuclear periphery by lamin B1. The histone chaperone ARTX is also recruited to IFI16 filaments, and the absence of IFI16 leads to diminished vDNA silencing. In accord with this, the absence of IFI16 leads to increased RNA Pol II recruitment to RCs, whereas cells capable of forming IFI16 filaments exhibit lower RNA Pol II density in all RCs. These results link IFI16-mediated restriction to IFI16 filament formation. This suggests that this subnuclear assembly of IFI16, the ND10 proteins, and the lamina plays a central role in the viral restriction pathway.

### IFI16, PML, and Sp100 assemble on DNA, with IFI16 as the nucleating component.

The domain structure of IFI16 with two DNA-binding HIN domains, previous studies on IFI16 filaments with DNA *in vitro* (12), and our immunofluorescence results argue that IFI16 is the component that binds directly to viral DNA. IFI16 filaments appear very thin and smooth, while PML and Sp100 filaments have a rougher appearance. This suggests that PML and Sp100 are not completely coating the vDNA. In addition, we found that IFI16 is the *sine qua non* for filamentous aggregation of the protein components. Sp100 and ATRX were still recruited to RCs in the absence of IFI16 but failed to form filamentous structures. Our results do not allow us to formally deduce direct interaction between selected proteins, but strongly suggest that IFI16, PML, and Sp100 and possibly lamin B1 are actually forming a multiprotein complex. The IFI16 filaments that we observed are susceptible to DNase digestion; therefore, it is likely they are based on a DNA structure. Second, viral DNA synthesis is required for their formation; therefore, the simplest model is that they represent IFI16 binding to progeny viral DNA strands. Consistent with this, some of the filaments approach the theoretical length of an extended HSV DNA molecule.

### Evidence for a role for IFI16 filaments in restriction of HSV and action in *trans*.

At least two lines of evidence connect the IFI16 filaments to the restriction activity exerted by IFI16 on HSV ICP0^−^ mutant gene expression and replication. First, when we measured the proportion of cells of different types that formed IFI16 filaments and the level of viral restriction by IFI16, we found a strong correlation between filament formation and ability to restrict viral replication in four different types of human cells. Furthermore, filaments formed by 6 hpi, and IFI16 increased the H3K9me3 heterochromatin mark on viral chromatin by 6 hpi. These two observations link the IFI16 filaments to restriction both kinetically as well as functionally.

Our results support the hypothesis that IFI16 oligomerizes on nascent viral DNA. However, we and others have shown that modified nucleoside labeling of nascent viral DNA using bromodeoxyuridine (BrdU), 5-ethynyl-2′-deoxycytidine (EdC), or 5-ethynyl-2′-deoxyuridine (EdU) correlates with ICP8 immunostaining throughout the replication compartment (23, 38). We found that IFI16 filaments were within replication compartments but did not colocalize with much of the ICP8, arguing that only a fraction of total newly synthesized vDNA is covered with IFI16. We confirmed that RCs are constituted of colocalizing foci of ICP8 and newly synthesized EdC-labeled vDNA; however, no filamentous EdC-labeled signal could be detected (results not shown).

The restrictive effect of IFI16 could be due in part to its binding to input viral DNA and preventing its transcription, leading to reduced expression of all viral genes. However, this does not seem to explain all of our results. In IFI16^+^ cells where viral gene expression occurs and replication compartments form, all of the replication compartments show reduced levels of elongating Pol II (Fig. 4B). Therefore, IFI16 seems to be affecting Pol II recruitment to and activity in replication compartments throughout the nucleus. This leads to the question of how IFI16 can restrict the 7134 virus despite binding to at best only a subset of progeny genomes. We speculate that IFI16 serves as a recruiting platform for other restriction factors like ATRX and has the capability to exert restrictive functions in *cis* and *trans*. Evidence for this is provided by our analysis of RNA Pol II recruitment to replication compartments. The absence of IFI16 leads to a uniform increase of RNA Pol II recruitment to early RCs, while this was not the case in cells in which IFI16 filament formation was possible. RCs formed in the nuclei of control cells exhibited similar RNA Pol II signal intensities, independent of the presence of IFI16 filaments in a single compartment. This argues that IFI16 filaments can diminish RNA Pol II recruitment to RCs in *trans*.

### IFI16 DNA filaments with associated host factors show the properties of a nuclear SMOC.

**FIG 7:**
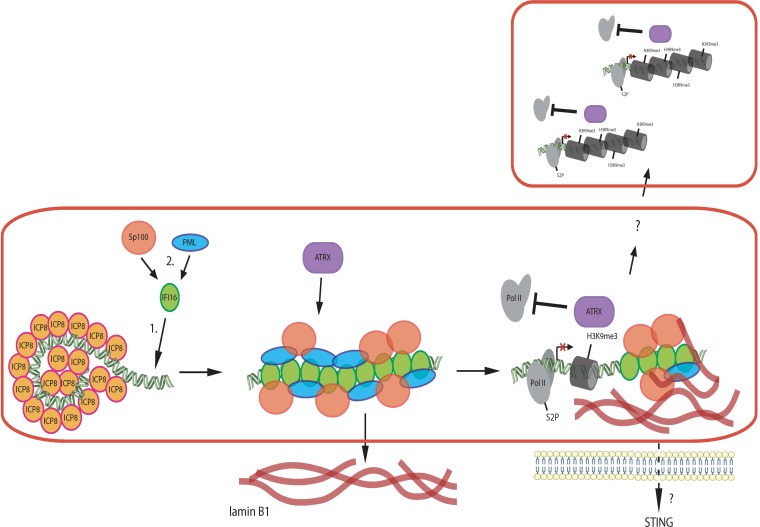
Restrictosome working model. IFI16 assembles on progeny viral DNA molecules and recruits other restriction factors, including PML, Sp100, and ATRX. These factors together exert a silencing effect in *cis* on the viral genome. In addition, a *trans*-acting silencing appears to occur on other viral genomes in the same and different replication compartments. ATRX is only partially localized to IFI16 filaments, so it may be part of the *trans*-acting silencing mechanism.

Several steps in the interaction of IFI16 interaction with HSV-infected cells have been documented. First, IFI16 has been shown to localize to parental genome complexes at the periphery of the nucleus (5, 11, 19, 21). Second, IFI16 later localizes to numerous foci that are coincident with HSV ICP0 protein and is then degraded (5). In cells infected with ICP0^−^ mutant viruses, IFI16 is not degraded and localizes to structures within replication compartments (5, 19; this report). We have shown that IFI16 decreases transcript levels from both input and progeny viral DNA (18); thus, there may be different mechanisms for restriction of gene expression at these different steps of IFI16 function. In this study, we defined the potential role of IFI16 filaments in reducing RNA Pol II levels in replication compartments throughout the infected cell nucleus. We observed that IFI16 reduces RNA Pol II recruitment to RCs, and there is no evidence that Pol II levels are different in different compartments depending on whether the RC has an IFI16 filament or not. Similarly, the distribution of Pol II appears to be uniform throughout the RCs, with no evidence of reduced Pol II near IFI16 filaments. Therefore, the IFI16 filaments appear to be acting in *trans* to reduce Pol II recruitment throughout the RCs. We hypothesize that the IFI16-based filaments are signaling platforms and propose to call them restrictosomes. A working model of the restrictosome is shown in Fig. 7.

Possible mechanisms for restrictosome signaling include both (i) Pol II modification and degradation and (ii) epigenetic modification of viral chromatin. The IFI16 filament structures could promote modification of Pol II, such as loss of serine 2 phosphorylation (39) or degradation (35). Thus, IFI16 could be causing loss of the RNA polymerase II holoenzyme large subunit or loss of critical modifications. Similarly, the IFI16 filament structures could be (i) activating a phosphatase that diffuses throughout the nucleus to dephosphorylate histones and cause heterochromatin formation, (ii) activating histone methyltransferases that diffuse through the nucleus and add heterochromatic marks, or (iii) activating demethylases that diffuse through the nucleus and remove euchromatic histone methylation marks. ATRX is only partially localized to IFI16 filaments; thus, ATRX could be binding to IFI16 filaments and translocating to other viral DNA molecules to silence viral gene expression. We have shown recently that ATRX plays a role in maintaining heterochromatin on viral DNA from 4 to 8 hpi (21); thus, ATRX could be one of the effectors activated by the IFI16 filaments.

In summary, we have defined a novel mechanism of epigenetic silencing of viral DNA after it has replicated in the nucleus of infected cells. We propose that the cellular restriction factor IFI16 binds to a newly replicated viral DNA genome and binds cooperatively to coat the molecule. Other host proteins are incorporated onto the filament, and the filament structure promotes signaling throughout the nucleus to epigenetically silence viral DNA in *trans*. Further studies are needed to identify the full protein composition of this new infected cell nuclear structure, which we propose to call a “restrictosome,” and to provide more information about the mechanism(s) by which this novel mechanism(s) functions.

## MATERIALS AND METHODS

### Cell culture, viruses, and infections.

Culture of HFF, U2OS, HeLa, and NOK cells was performed as described previously (18). Tert-HF cells were treated like HFF cells. The HSV-1 7134 ICP0-null virus and the respective rescued virus 7134R (40) were propagated and titrated in parallel on U2OS cells (41). For infections, viruses were diluted in phosphate-buffered saline (PBS) containing 0.1% (wt/vol) glucose and 1% (vol/vol) bovine calf serum (BCS). Infections were carried out at the indicated MOI for 1 h at 37°C, and then the inoculum was replaced with Dulbecco’s modified Eagle’s medium (DMEM) containing 1% BCS, and the cells were kept at 37°C for the time indicated. If not indicated otherwise, cells were infected with the 7134 ICP0-null virus at an MOI of 5 and fixed or harvested at 6 hpi.

To determine the effects of inhibition of viral DNA replication, HFF cells were infected with HSV-1 7134 virus, in the presence of 400 µg/ml phosphonoacetic acid (PAA) and 10 mM HEPES (pH 7.4). Cells were incubated for 1 h at 37°C and then overlaid with DMEM containing 0.1% BCS, 400 µg/ml PAA, and 10 mM HEPES (pH 7.4) and harvested at the time indicated.

### IFI16 CRISPR-Cas9 knockout tert-HF cell lines.

Telomerase-immortalized HF (Tert-HF) cells, derived from HFF cells (42), were generously provided by Robert Kalejta (University of Wisconsin). Double-stranded DNA oligonucleotides encoding IFI16-specific gRNAs (18) were ligated into BsmBI-digested lentiCRISPR v2 lentivirus vector (43). Sequence-verified lentiCRISPR v2 plasmids expressing Cas9 alone or coexpressing guide RNA (gRNA) 1 or 4 were cotransfected with pLenti packaging mix (pVSV-G and psPAX2) into HEK293T cells. Supernatants were harvested at 24 and 48 h posttransfection and filtered through a 0.45-μm-pore filter. All of the supernatant from each construct was added to one well containing 1 × 10^5^ Tert-HF cells plated in a 6-well plate. The medium was changed at 24 hpi, and cells were put under puromycin selection (4 μg/ml) at 3 days postinfection and subsequently expanded.

### Immunofluorescence and image analysis.

Cells were grown, fixed with formaldehyde, permeabilized, and incubated with the indicated antibodies (Table 1) as described before (8). All images except Fig. 2A, Fig. 1F, and Fig. S1E to G were acquired with an OMX V4 Blaze (GE Healthcare) structured illumination superresolution microscope with an Olympus 60×/1.42 Plan Apo objective. Spherical aberration was minimized using matching immersion oil (usually a refractive index of 1.518). Alexa Fluor 488 fluorescence was excited with a 100-mW 488-nm laser and collected with a 528/48m emission filter (Omega). Alexa Fluor 594 was excited with a 100-mW 568-nm laser and collected with a 609/37m emission filter (Omega). Images were acquired with a PCO.edge sCMOS camera controlled with DeltaVision OMX software. z-stacks were collected with a step size of 0.125 μm, for a total thickness of ∼2.5 µm. For each z-section, 15 raw images (three rotations with five phases each) were acquired. Superresolution images were computationally reconstructed from the raw data sets with a channel-specific, measured optical transfer function and a Wiener filter constant of 0.001 using CUDA-accelerated 3D-SIM reconstruction code based on Gustafsson et al. (44). TetraSpeck beads (Thermo Fisher) or a nano-grid control slide (GE) was used to measure axial and lateral chromatic misregistration, and experimental data sets were registered using the imwarp function in MATLAB (MathWorks). z-series are displayed as maximum intensity z-projections or single slices as indicated, and brightness and contrast and pseudocolor were adjusted using Fiji software. Pearson coefficients were calculated with the Fiji plugin Coloc2. Area, integrated fluorescence intensity, and length measurements were done with Fiji. Three-dimensional (3D) iso-surface rendering, animations, and 3D colocalization analysis were done with Bitplane Imaris. The remaining images were acquired with a Zeiss Axioplan widefield fluorescence microscope. Deconvolution was done in an iterative fashion with Zeiss AxioVision Software. Additional postprocessing was performed with Fiji as described above.

**TABLE 1:** Antibodies used in this study

Antibody specificity^*a*^	Source or reference
IFI16, mouse	Abcam ab50004
PML, rabbit	Bethyl A301-167A
PML, mouse	Abcam ab96051
Sp100, rabbit	Proteintech 11377-1-AP
Sp100, mouse	Millipore MABC1037
Lamin B1, rabbit	Abcam ab16048
ATRX, rabbit	Abcam ab97508
Pol II, unphosphorylated CTD, rabbit	Abcam ab26721
Pol II phosphor S2, rabbit	Abcam ab5095
GAPDH, mouse	Abcam ab8245
ICP8, rabbit	47
39S ICP8, mouse	48
ICP27, mouse	Abcam ab31631
ICP4, mouse	Abcam ab6514
H3K9me3, rabbit	Abcam ab8898
H3, rabbit	Abcam ab1791
Anti-mouse secondary for IF, Alexa 488, goat	Abcam ab150113
Anti-rabbit secondary for IF, Alexa 594, goat	Abcam 150080
Anti-mouse secondary for WB, HRP, horse	Cell Signaling 7076S
Anti-rabbit secondary for WB, HRP, goat	Cell Signaling 7074S

a GAPDH, glyceraldehyde-3-phosphate dehydrogenase; IF, immunofluorescence; WB, Western blotting; HRP, horseradish peroxidase.

### In-cell nuclease digestion.

We adapted a protocol from Randall and Dinwoode (45). HFF cells on coverslips were prefixed with 0.5% formaldehyde in PBS for 30 min, washed with PBS, permeabilized in 0.5% NP-40 for 10 min, and washed with PBS again. Coverslips were incubated with 20 U of RNase-free DNase (NEB) or DNase-free RNase for 30 min at 37°C. The coverslips were then washed again, fixed in 2% formaldehyde in PBS for 10 min, and stained with the appropriate antibodies.

### ChIP.

ChIP was performed as described previously (46), using Cas9 control cell and no. 4 IFI16 ko HF cell lysates with antibodies specific for H3 and H3K9me3 (Table 1). Relative quantification of DNA was done with qPCR (Fast SYBR Green reagents) with primers specific for the *ICP4*, *ICP8*, and *ICP27* genes (18).

### Depletion of proteins.

siRNAs specific for IFI16, PML, or nontargeting siRNA were purchased from Dharmacon (18). siRNA transfection in HFF cells was done using RNAi Max (Invitrogen) according to the manufacturer’s specifications.

### Statistical analysis.

All statistical analyses were performed with GraphPad Prism 7, except for Fig. 1E. For single-column comparisons, *t* tests were run. Statistical significance is indicated by asterisks as follows: *, *P* < 0.05; **, *P* < 0.01; ***, *P* < 0.001; ****, *P* < 0.0001. For Fig. 1C, a Mann-Whitney-Wilcoxon test was done with R.

### Edge of plaque immunofluorescence analysis.

HFF cells were seeded on coverslips and infected with 7134 virus at low MOI (0.1 to 1 PFU/cell). The inoculum was replaced at 1 hpi with DMEM containing 1% BCS and 0.32% human IgG. After plaque formation, cells were fixed and processed for immunofluorescence analysis as described above.
