# Supplementary material for: Role for a Filamentous Nuclear Assembly of IFI16, DNA, and Host Factors in Restriction of Herpesviral Infection
Source: mBio. 2019 Jan 22;10(1):e02621-18. doi: 10.1128/mBio.02621-18 (PMC6343039; doi:10.1128/mBio.02621-18)
Supplement: FIG S1 [file mBio.02621-18-sf001.pdf]

Suppl. Fig. 1

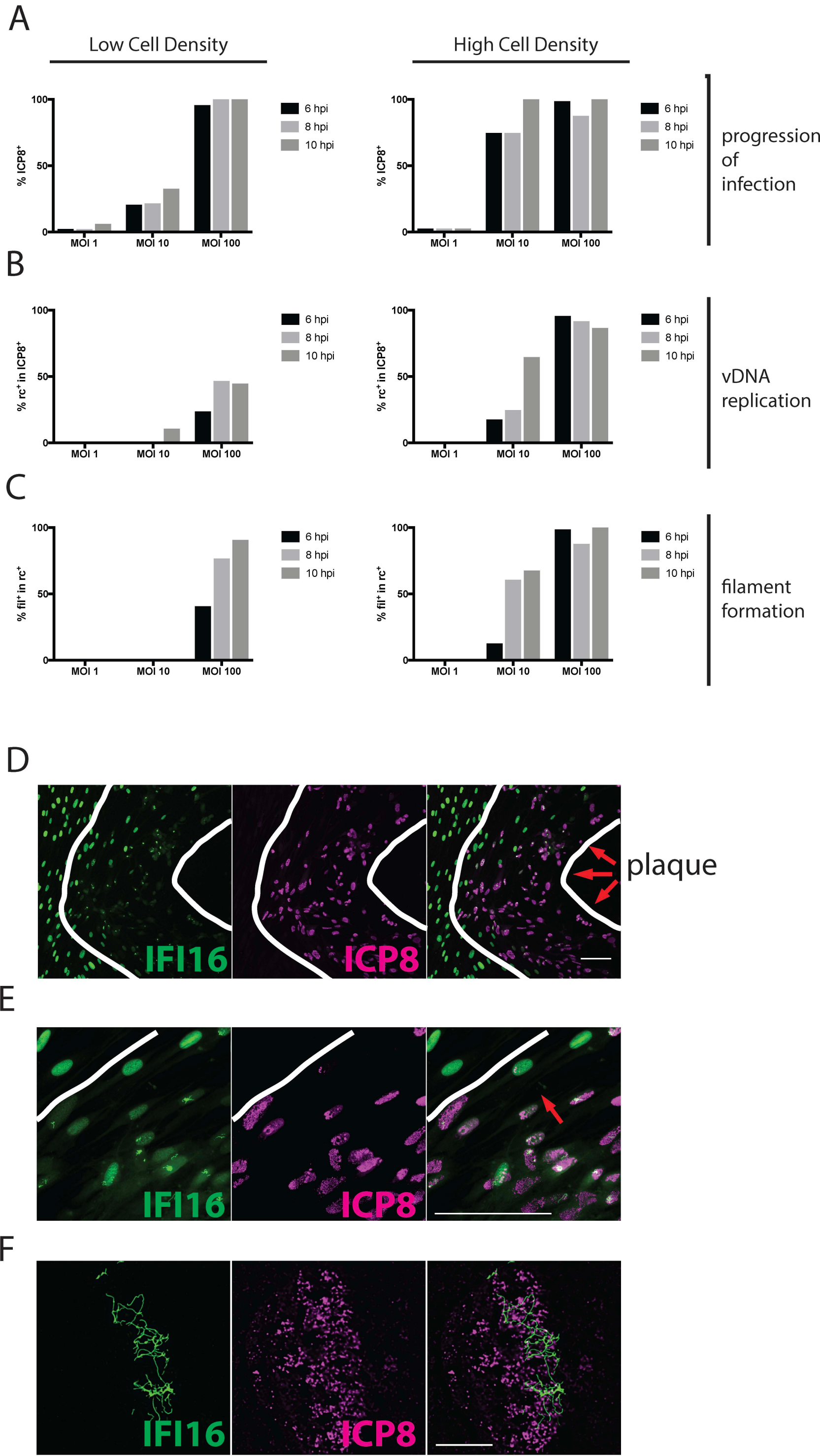
A
Low Cell Density
High Cell Density
% ICP8+
100
50
0
MOI 1
MOI 10
MOI 100
6 hpi
8 hpi
10 hpi
progression of infection
B
% rc+ in ICP8+
vDNA replication
C
% fil+ in rc+
filament formation
D
IFI16
ICP8
plaque
E
F
