# Supplementary figures and images for: Role for a Filamentous Nuclear Assembly of IFI16, DNA, and Host Factors in Restriction of Herpesviral Infection
Source: mBio. 2019 Jan 22;10(1):e02621-18. doi: 10.1128/mBio.02621-18 (PMC6343039; doi:10.1128/mBio.02621-18)

Suppl. Fig. 2

HFF

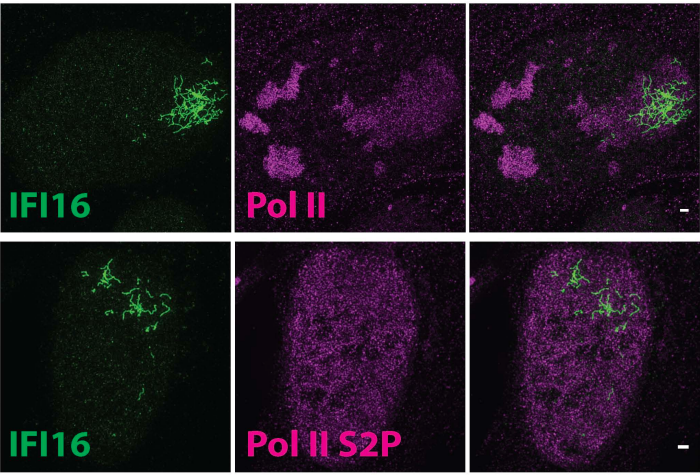

Supplement: FIG S2 [file mBio.02621-18-sf002.pdf]

Suppl. Fig. 3

A

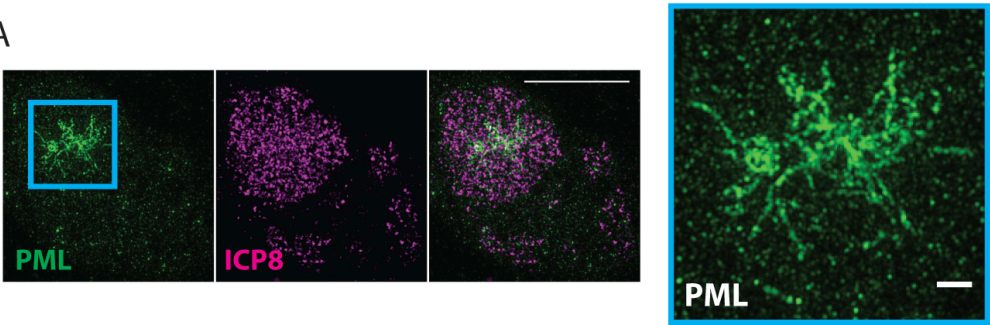

B

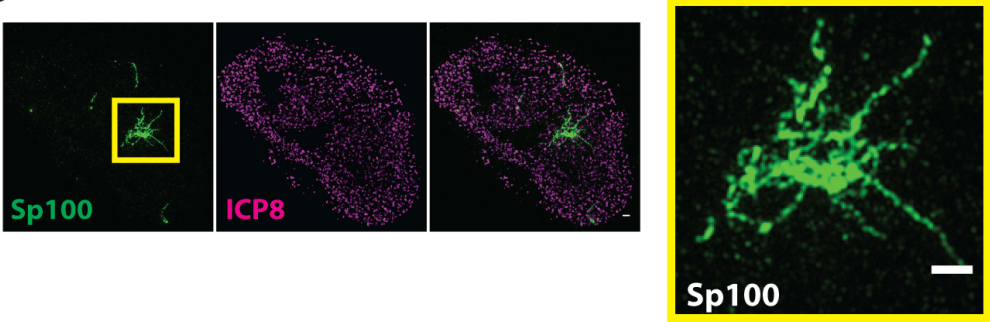

C

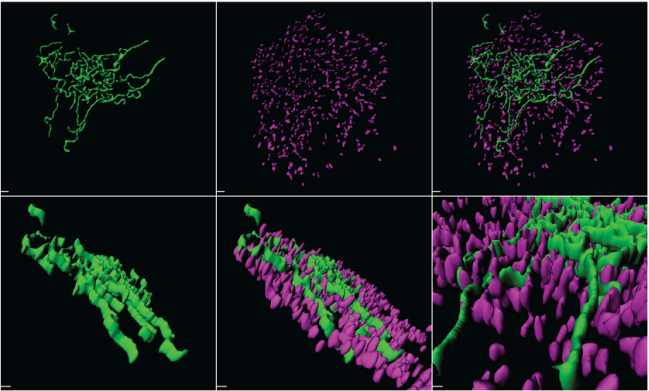

Supplement: FIG S3 [file mBio.02621-18-sf003.pdf]

Suppl. Fig. 4

A

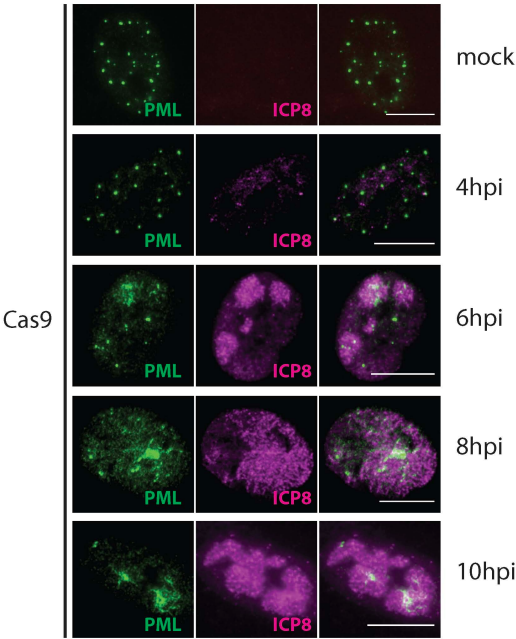

B

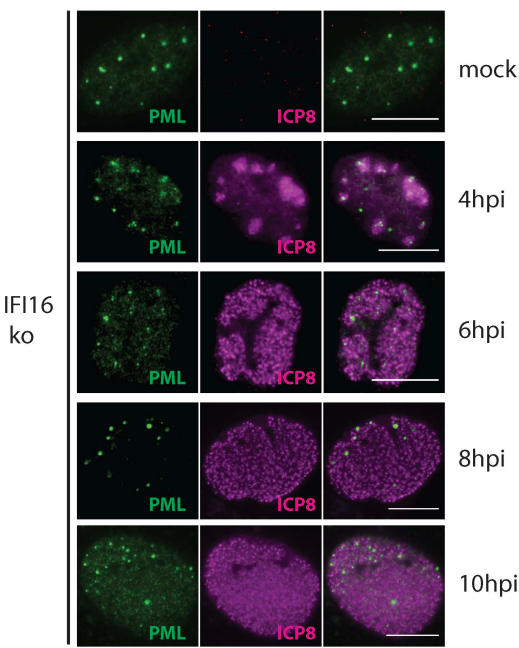

C

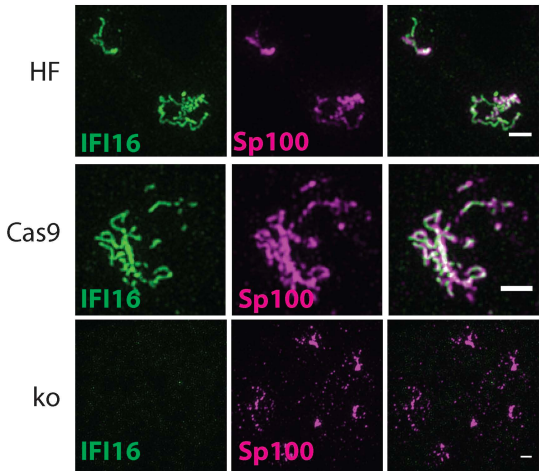

Supplement: FIG S4 [file mBio.02621-18-sf004.pdf]
